# Thoracic pedicle classification determined by inner cortical width of pedicles on computed tomography images: its clinical significance for posterior vertebral column resection to treat rigid and severe spinal deformities—a retrospective review of cases

**DOI:** 10.1186/1471-2474-15-278

**Published:** 2014-08-13

**Authors:** Ying Zhang, Jingming Xie, Yingsong Wang, Ni Bi, Zhi Zhao, Tao Li

**Affiliations:** Department of Orthopaedics, 2nd Affiliated Hospital of Kunming Medical University, #374 Dianmian Road, Kunming, Yunnan Province 650101 P.R. China

**Keywords:** Scoliosis, Thoracic vertebrae, Pedicle, Computed tomography, Posterior approach, Vertebral column resection

## Abstract

**Background:**

Posterior vertebral column resection (PVCR) is an effective alternative for treating rigid and severe spinal deformities. Accurate placement of pedicle screws, especially apically, is crucial. As morphologic evaluations of thoracic pedicles have not provided objective criteria, we propose a thoracic pedicle classification for treating rigid and severe spinal deformities.

**Methods:**

A consecutive series of 56 patients with severe and rigid spinal deformities who underwent PVCR at a single institution were reviewed retrospectively. Altogether, 1098 screws were inserted into thoracic pedicles at T2-T12. Based on the inner cortical width of the thoracic pedicles, the patients were divided into four groups: group 1 (0–1.0 mm), group 2 (1.1–2.0 mm), group 3 (2.1–3.0 mm), group 4 (≥3.1 mm). The proportion of screws accurately inserted in thoracic pedicles for each group was calculated. Statistical analysis was also performed regarding types of thoracic pedicles classified by Lenke *et al.* (SPINE 35:1836-1842, 2010) using a morphological method.

**Results:**

There were statistically significant differences in the rates of screws inserted in thoracic pedicles between the groups (*P* < 0.008) except groups 3 and 4 (*P >* 0.008), which were then combined. The accuracies for the three new groups were 35.05%, 65.34%, and 88.32%, respectively, with statistically significant differences between the groups (*P <* 0.017). Rates of screws inserted in thoracic pedicles classified by Lenke *et al.* (SPINE 35:1836-1842, 2010) were 82.31%, 83.40%, 80.00%, and 30.28% for types A, B, C, and D, respectively. There was no statistically significant difference (*P >* 0.008) between these types except between type D and the other three types (*P <* 0.008).

**Conclusions:**

The inner cortical width of thoracic pedicles is the sole factor crucial for accurate placement of thoracic pedicle screws. We propose a computed tomography-based classification of the pedicle’s inner cortical width: type I thoracic pedicle: absent channel, inner cortical width of 0–1 mm; type II: presence of a channel of which type IIa has an inner cortical width of 1.1–2.0 mm and type IIb a width of ≥2.1 mm. The proposed classification can help surgeons predict whether screws can be inserted into the thoracic pedicle, thus guiding instrumentation when PVCR is performed.

**Electronic supplementary material:**

The online version of this article (doi:10.1186/1471-2474-15-278) contains supplementary material, which is available to authorized users.

## Background

The combined use of posterior vertebral column resection (PVCR) and pedicle instrumentation has allowed greater deformity correction and overall spinal balance than are achieved with conventional approaches. In recent years, reports on the clinical use of this technique have gradually increased. In particular, PVCR may be the best procedure for correcting severe (Cobb angle >100°), angular, and rigid (flexibility <10%) spinal deformities with significant decompensation in the coronal or sagittal plane. Studies have indicated, however, that severe spinal deformities are often accompanied by congenital or secondary pedicle deformities that might be associated with a high risk of malpositioned pedicle screws and vascular or neurological complications [1].

When PVCR is performed to treat severe spinal deformities, the most crucial step is to insert the screw within the pedicle. Factors affecting the accuracy of thoracic pedicle screw insertion have been well studied. However, most studies focused on the morphology of the pedicles [2–5], which gives little help to surgeons for predicting the accuracy and guiding the procedure of thoracic pedicle screw placement. There are few studies on the correlation between the diameter of the thoracic pedicle channel and the accuracy of thoracic pedicle screw placement.

The present study included a consecutive series of 56 patients with severe and rigid spinal deformities undergoing PVCR at a single institution between October 2004 and July 2010. A total of 1098 thoracic pedicles were studied, statistically analysed, and classified to demonstrate the clinical significance of treating severe and rigid spinal deformities with PVCR.

## Methods

A consecutive series of 56 patients with severe and rigid spinal deformities who underwent PVCR at a single institution between October 2004 and July 2010 were included in the study. The Scientific Committee of the Second Affiliated Hospital of Kunming Medical University, Yunnan Province, China gave academic, institutional, and ethical approval of the research protocol and procedures. All participants were provided with full information about the study’s procedures and aims and gave informed consent to participate in the study, according to the principles of medical ethics of the World Health Organization and the Helsinki Declaration. All patients gave informed consent for their radiological images and information to be published. There were 20 male and 36 female patients with an average age of 21.5 years (range 10–48 years) at the time of the surgeries. The patients had a main curve with average Cobb angles of 104.6° ± 35.9° and 94.8° ± 27.7° in the coronal and sagittal planes, respectively. One surgeon performed all of the operations. The screws were inserted with the free-hand technique using a 2-mm hand drill. A total of 1098 thoracic pedicle screws were inserted at T2-T12. The average follow-up was 48 months.

### Measurement methods and evaluation criteria

The patients underwent standing anteroposterior and lateral radiography before and after the surgery. The Cobb angle of the curve was measured to evaluate spinal deformities in the coronal and sagittal planes. All thoracic pedicles to be instrumented were scanned preoperatively using Philips 256 computed tomography (CT) equipment (200 mAs/120 kV, thickness of scanning and reconstruction 0.8 mm, scan interval 0.625 mm). Volume rendering (VR) and multiplanar reconstruction (MPR) were also performed. The inner cortical width of thoracic pedicles was measured horizontally at the narrowest point of each thoracic pedicle. Postoperatively, axial CT imaging and reconstruction of the thoracic pedicles with a thickness of 0.8 mm was performed parallel to the axis of the inserted screw. A senior doctor evaluated the accuracy of the thoracic pedicle screws’ placement. Penetration of the internal and external cortical walls of the thoracic pedicles was measured in millimetres and classified into three degrees: 0 (no penetration); 1 (≤2 mm penetration of the pedicle’s cortical wall); 2 (>2 mm penetration of the pedicle’s cortical wall). The accuracy of thoracic pedicle screw placement was confirmed using the following criteria: (1) Pedicle screw trajectory during the surgery was accomplished with a 2-mm hand drill. A 1-mm self-made pedicle trajectory probe was used to confirm that the five walls of the channel were intact. (2) Postoperative CT scanning of the instrumented thoracic pedicles was performed to confirm that the axis of each screw was completely within the pedicle. Degree 0 and 1 penetration of the thoracic pedicle indicated accurate placement of the screw. (3) No complications relevant to thoracic pedicle screws occurred postoperatively, such as nerve root irritation or spinal cord complications.

### Measurement of the inner cortical width of thoracic pedicles, pedicle classification, and statistical analysis

The inner cortical width of the thoracic pedicles was measured in millimeters, and the pedicles were then classified into four groups based on their inner cortical width: group 1 (0–1 mm), group 2 (1.1–2.0 mm), group 3 (2.1–3.0 mm), group 4 (≥3.1 mm). The placement accuracy of the thoracic pedicle screws in the groups was calculated. The χ^2^ test was used for comparison of the accuracy of thoracic pedicle screws between the groups. Statistical analysis was performed using SPSS software (SPSS, Chicago, IL, USA). Statistical significance was defined as *P* < 0.05 for overall comparisons and as *P* < 0.008 for comparisons between the four groups.

The four groups were then classified into three new groups: new group 1 (0–1 mm), new group 2 (1.1–2.0 mm), new group 3 (≥2.1 mm). The χ^2^ test was again used to compare the accuracy of thoracic pedicle screw placement in these groups. Statistical analysis was performed using SPSS. Statistical significance was defined as *P* < 0.05 for overall comparisons and as *P* < 0.017 for comparisons between the three new groups. The accuracy of thoracic pedicle screw placement was also calculated based on the morphological classification of pedicles [6], and the χ^2^ test was used for comparison of the accuracy of thoracic pedicle screw placement among different morphological types. Statistical analysis was performed using SPSS. Statistical significance was defined as *P* < 0.05 for overall comparisons and as *P* < 0.008 for comparisons between different types.

## Results

### Statistical analysis of accurate screw placement in thoracic pedicles determined by the inner cortical width of pedicles

Among the 1098 thoracic pedicles in the study, 826 (75.23%) were accurately instrumented with pedicle screws. Among the original four pedicle groups, 68 screws were accurately inserted into 194 thoracic pedicles in group 1 (0–1.0 mm inner thoracic pedicle width), 115 screws in 176 thoracic pedicles in group 2 (1.1–2.0 mm), 304 screws in 352 thoracic pedicles in group 3 (2.1–3.0 mm), and 339 screws in 376 thoracic pedicles in group 4 (≥3.0 mm). The accuracies of thoracic pedicle screw placement were 35.05%, 65.34%, 86.36%, and 90.16% in groups 1, 2, 3, and 4, respectively. Statistical analysis was performed to compare the accuracy of thoracic pedicle screw placement among these groups. The differences were statistically significant (*P* < 0.008) between all of the groups except between groups 3 and 4 (*P* > 0.008) (Table 1).

**Table 1:** **Statistical analysis on accurately instrumented thoracic pedicles determined by inner pedicle cortical width**

Inner cortical width of thoracic pedicles (mm)	No. of thoracic pedicles instrumented	No. of thoracic pedicles accurately instrumented	Accuracy (%)
0–1.0	194	68	35.05
1.1–2.0	176	115	65.34
2.1–3.0	352	304	86.36
≥3.1	376	339	90.16
Total	1098	826	75.23

### Accurate rate of screws inserted in three new groups of thoracic pedicles and in four types of thoracic pedicles determined by the morphological classification method

**Figure 1:**
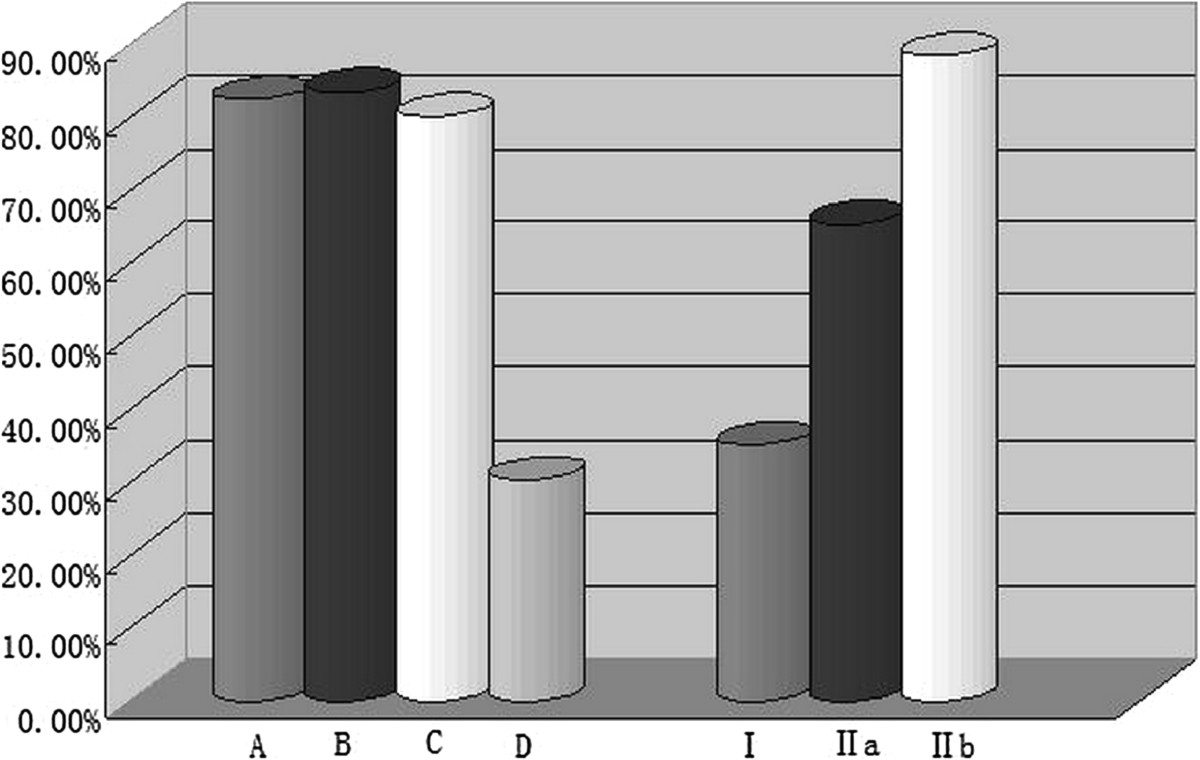
**Accuracy of thoracic pedicle screw placement for different groups/types of thoracic pedicles determined by two classification methods.**

When the original four groups of thoracic pedicles being classified into three new groups determined by the inner cortical width, the accuracy of thoracic pedicle screw placement was as follows: 68 screws were accurately inserted into 194 thoracic pedicles in new group 1 (0–1.0 mm), 115 screws in 176 thoracic pedicles in new group 2 (1.1–2.0 mm), 643 screws in 728 thoracic pedicles in new group 3 (2.1–3.0 mm). The accuracies of thoracic pedicle screw placement were 35.05%, 65.34%, and 88.32% in new groups 1, 2, and 3, respectively. The accuracy was then compared among these new groups and the difference was found to be statistically significant among all of the new groups (*P* < 0.017) (Table 1, Figure 1).

**Figure 2:**
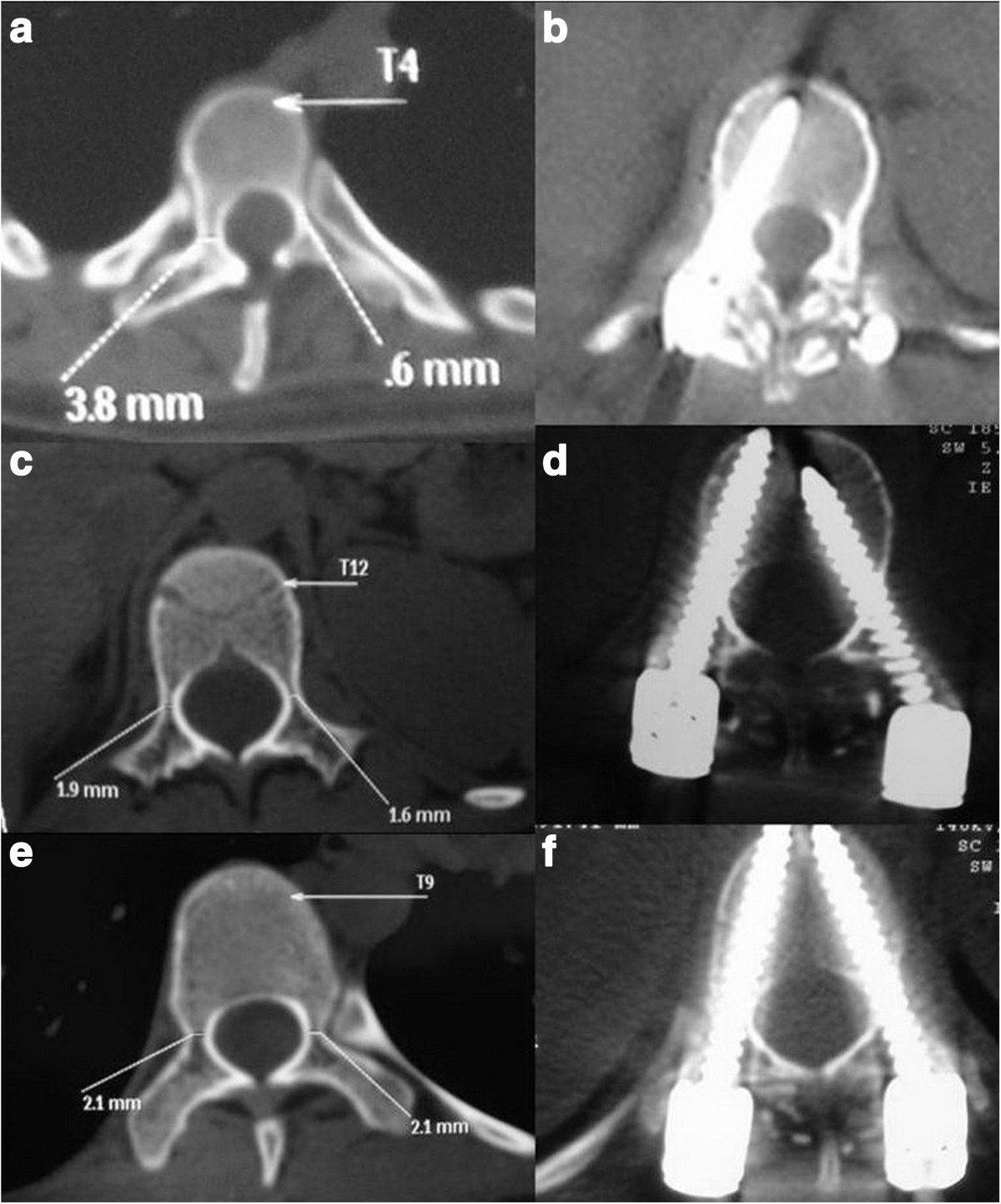
**Preoperative and postoperative computed tomography images of pedicle classification determined by the inner cortical width. (a)** Preoperative type I pedicle. **(b)** Postoperative type I pedicle. **(c)** Preoperative type IIa pedicle. **(d)** Postoperative type IIa pedicle. **(e)** Preoperative type IIb pedicle. **(f)** Postoperative type IIb pedicle.

**Figure 3:**
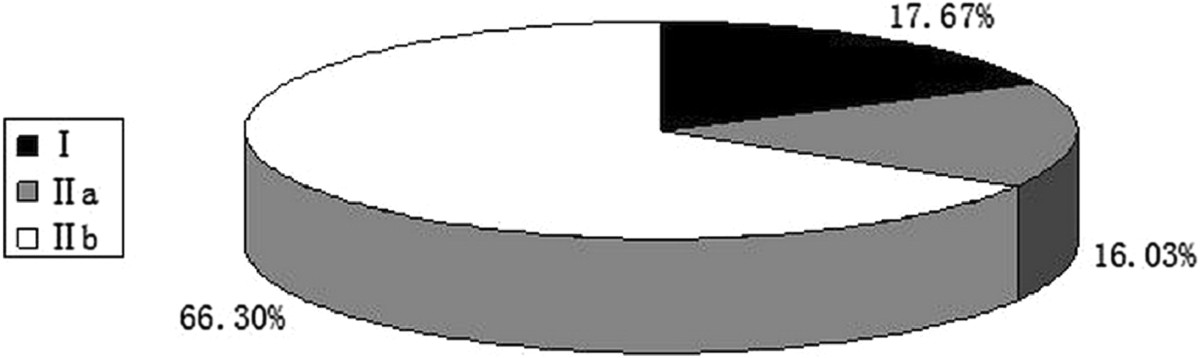
**Composition of the various thoracic pedicles studied.**

The thoracic pedicles studied were then reclassified into four types based on the classification method proposed by Lenke *et al.*[6]. The accuracies for thoracic pedicle screw placement in Lenke *et al*’s types A, B, C, and D were 82.31%, 83.40%, 80.00%, and 30.28%, respectively. Statistical analysis indicated that there were no statistically significant differences in the accuracy of thoracic pedicle screw placement between these types (*P* > 0.008), except between type D and the other three types (*P* < 0.008) (Table 2, Figure 1).Statistical analysis of the aforementioned data indicated that, with or without the channel in thoracic pedicles, the accuracy of thoracic pedicle screw placement varied significantly. Among the thoracic pedicles with a channel, there was a statistically significant difference in the accuracy of thoracic pedicle screws between thoracic pedicles with an inner cortical width of 1.1–2.0 mm and those whose channel was ≥2.1 mm. This difference demonstrated that the accuracy of thoracic pedicle screw placement was closely correlated with (1) whether the thoracic pedicle had a channel and (2) its inner cortical width. Hence, we propose a classification method to use as a guide in clinical practice for predicting whether a screw can be accurately inserted into a thoracic pedicle. The classification is based on determining the cortical width of the inner pedicle on CT images: Type I includes thoracic pedicles that have no channel and an inner pedicle width of 0–1 mm. Type II includes thoracic pedicles that have a channel. The type II thoracic pedicles would then be subclassified as type IIa (with an inner cortical width of 1.1–2.0 mm) or type IIb (with an inner cortical width ≥2.1 mm) (Figure 2).Among the 1098 thoracic pedicles, there were 194 type I pedicles, accounting for 17.67% of the total thoracic pedicles studied. The other 904 pedicles were type II, of which 176 (16.03%) were type IIa, and 728 were type IIb (66.30%) (Figure 3).

**Table 2:** **Accuracy of thoracic pedicle screws in four types of thoracic pedicles determined by the morphological classification method of Lenke** ***et al.***[6]

Type of thoracic pedicles	No. of thoracic pedicles instrumented	No. of thoracic pedicles accurately instrumented	Accuracy of thoracic pedicles instrumented (%)
A	441	363	82.31
B	235	196	83.40
C	280	224	80.00
D	142	43	30.28
Total	1098	826	75.23

## Discussion

Common practice for surgical treatment of severe and rigid spinal deformities includes anterior release and fusion, one- or two-stage posterior fixation combined with spinal osteotomy, vertebral column resection (VCR), etc.. These treatments to correct and fix spinal deformities have been well accepted by spine surgeons. However, for severe, rigid, and angular spinal deformities, it is not easy to obtain desirable correction results using these approaches.

In recent years, some authors have reported the clinical use of posterior VCR (PVCR) for correcting spinal deformities in patients with spinal scoliosis or kyphosis [7, 8]. In particular, for patients with severe (Cobb angle >100°), angular (the angle changed significantly through a few spinal segments), and rigid (flexibility <10%) spinal deformities that are accompanied with significant coronal or sagittal decompensation, PVCR will be the best alternative [9]. Severe spinal deformities are frequently accompanied by congenital or secondary pedicle deformities that are often located in the thoracic spine. When PVCR is performed to correct the deformities, the most crucial step is to insert screws in the pedicles, particularly pedicles in the apical region of the main curve in the thoracic spine.

Many factors have an impact on the accuracy and safety of thoracic pedicle screw placement. The rate of thoracic pedicle screw malpositioning during correction of spinal deformities is as high as 3.0–44.2%, and the rate complications related to pedicle screws is 0.9% [10–12]. For surgeons, especially in the process of applying PVCR, the preoperative reliable observation and analysis of each pedicle will be very helpful for the accurate insertion of screws and the final decision of fusion segments. Therefore, a quantitative description method or criteria for pedicles will help the preoperative plan, shorten the duration of operation and decrease the risks being caused by screw insertion. However, up to date, there are only few reports on the correlation between the inner cortical width of thoracic pedicles and the accuracy of thoracic pedicle screw placement.

The present study included a consecutive series of 56 patients with severe and rigid spinal deformities who underwent PVCR at a single institution between October 2004 and July 2010. A total of 1098 thoracic pedicles instrumented at T2-T12 were reviewed. We have proposed criteria for establishing a thoracic pedicle classification based on the inner cortical width of thoracic pedicles as determined on CT scans, and believe these criteria can be of clinical significance.

### Selecting a measurement method to quantify relevant values for thoracic pedicles

When the accuracy of pedicle screw placement is evaluated, routine radiography is of little help to the surgeon to determine directly whether the pedicle has been penetrated. The accuracy of routine radiography for evaluating pedicle screw placement varies significantly, ranging from 10% to 83% [13]. Routine two-dimensional CT scans can be easily affected by metal artifacts and cannot objectively quantify pedicle penetration. Three-dimensional reconstruction of pedicles on multi-slice spiral CT images is of great importance. By rotating and cutting VR images at random, this method can derive values indicating the inner cortical width of pedicles that are very close to the actual transverse diameter of the pedicle isthmus. It is therefore considered the gold standard [14, 15]. It is certainly more reliable than others. Therefore, in the present study, the thoracic vertebrae of scoliotic patients were scanned, and thin-slice VR and MPR of the thoracic pedicles were performed.

Some studies indicated that the gaps between thoracic pedicles of the upper and middle thoracic vertebrae and dural sac were small, whereas the gap between the pedicles of the lower thoracic vertebrae and dural sac are as large as 2 mm [16]. Therefore, in the present study, when penetration of the internal and external osseous walls of thoracic pedicles was evaluated, it was measured in millimetres. On the basis of that evaluation, we proposed a method to classify thoracic pedicle penetration from the viewpoint of clinical significance. That is, the penetration of internal and external cortical walls of thoracic pedicles could be classified into three degrees: 0, no penetration; 1, thoracic pedicle penetration ≤2 mm; 2, thoracic pedicle penetration >2 mm. Degrees 0 and 1 penetration of thoracic pedicle walls indicate that the screws had been inserted into thoracic pedicles.

Whether the inner or outer cortical width of thoracic pedicles should be measured when studying the morphology of thoracic pedicles has been debated [3, 17–19]. In the present study, when measuring the cortical width of thoracic pedicles in the transverse plane, we measured the inner (rather than the outer) cortical width of thoracic pedicles because we believed that this method was more reliable for determining the pedicle screw diameter and for demonstrating the effects of thoracic pedicle instrumentation [20].

### Characteristics and clinical significance of quantification classification of thoracic pedicles determined by their inner cortical width on CT images

Along with the pedicle classification method of Lenke *et al.*[6], previous studies on pedicles focused on morphology. They lacked a quantification standard and were poorly consistent, making classification methods based on their findings rather subjective. Furthermore, such classifications gave little help to surgeons for predicting the accuracy of thoracic pedicle instrumentation with the free-hand technique before surgery. For all of these reasons, these classifications never gained popularity.

The study by Liljenqvist *et al.*[2] noted that the angulations of spinal deformities in the coronal plane and rotation of the vertebral body were not significantly correlated with the accuracy of thoracic pedicle screw placement, a finding that has been gradually accepted. In the present study, after having meticulously studied 1098 thoracic pedicles of scoliotic patients on CT images and analysed the data, we noted that there was a statistically significant difference in the accuracy of thoracic pedicle screws between pedicles with and those without a channel. In the groups of thoracic pedicles having a channel, there was statistically significant difference in the accuracy of thoracic pedicle screws between pedicles with an inner cortical width of 1.1–2.0 mm and those with a width of ≥2.1 mm. According to the morphological classification of thoracic pedicles proposed by Lenke *et al.*[6], there was a statistically significant difference in the accuracy of thoracic pedicle screws between their type D without a pedicle channel and the three other types (A–C) (*P* < 0.008). The statistical data of the current study and our clinical experiences indicated that, among many other factors affecting the accuracy and safety of thoracic pedicle screws, the angulations of the spinal deformities in the coronal plane and the rotation of the vertebral body only indirectly affected the accuracy and safety of thoracic pedicle screw placement by increasing the difficulty of the surgeries. The only crucial factors that had a direct impact on the accuracy and safety of thoracic pedicle screw placement and safety were (1) whether the pedicle had a channel and (2) the inner cortical width of the thoracic pedicles.

Therefore, we propose the following thoracic pedicle classification based on the inner cortical width of the thoracic pedicle on CT scans: type I thoracic pedicle, which has no channel and an inner cortical width of 0–1 mm; type II thoracic pedicle, which has a channel. Type II thoracic pedicles are then subclassified: type IIa, which has an inner cortical width of 1.1–2.0 mm, and type IIb, which has an inner cortical width of ≥2.1 mm. As the thoracic pedicle classification criteria have been quantified and the morphology of the thoracic pedicles is not taken into account, the consistency and reliance of this new thoracic pedicle classification method is an improvement. Comparisons on the accuracy of thoracic pedicle screws between the two classification methods showed that thoracic pedicle classification determined by the inner cortical width of the pedicle on CT images can help surgeons predict whether a screw could be accurately inserted into the thoracic pedicle—which was of clinical significance.

Regarding the distribution of different types of thoracic pedicle, types I, IIa, and IIb thoracic pedicles accounted for 17.67%, 16.03%, and 66.30%, respectively, of the total thoracic pedicles studied. The accuracies of pedicle screw placement in types I, IIa, and IIb thoracic pedicles were 35.05%, 65.34%, and 88.32%, respectively. Evaluation of thoracic pedicles prior to or during PVCR procedures is of significant importance. Type IIb pedicles of the thoracic vertebrae are suitable for screw insertion, and screw placement can be attempted in type IIa pedicles. In contrast, the accuracy of thoracic pedicle screw placement in type I pedicles is extremely low, particularly when the thoracic pedicles are adjacent to apical vertebrae or are above or below vertebrae that are to be resected. In these cases, PVCR should be selected cautiously or completely abandoned.

PVCR is an effective approach for treating severe spinal deformities. Whether the screw can be inserted into a morphologically abnormal pedicle is crucial for accurate pedicle screw placement. In the present study, we noted that the inner cortical width of the thoracic pedicles was the sole decisive factor for predicting the accuracy of thoracic pedicle screw placement. On that basis, we propose quantification classification criteria for thoracic pedicles that could help surgeons predict whether a screw could be inserted into a thoracic pedicle during PVCR, thus guiding the instrumentation of thoracic pedicles with the free-hand technique.

## Conclusions

The inner cortical width of thoracic pedicles was the sole factor crucial for predicting the accuracy of thoracic pedicle screw placement. We proposed a quantification classification method of thoracic pedicles based on determining the inner cortical width of pedicles using CT: Type I thoracic pedicle has no channel and an inner cortical width of the thoracic pedicle of 0–1 mm. Type II thoracic pedicle has a channel, which is subclassified into type IIa (with a thoracic pedicle having an inner cortical width of 1.1–2.0 mm) and type IIb (with a thoracic pedicle having an inner cortical width of ≥2.1 mm). The proposed thoracic pedicle classification method can help surgeons predict whether the screw can be inserted into a thoracic pedicle, thus guiding accurate thoracic pedicle instrumentation when PVCR is performed.

## Authors’ original submitted files for images

Below are the links to the authors’ original submitted files for images. Authors’ original file for figure 1 Authors’ original file for figure 2 Authors’ original file for figure 3
