# Supplementary figures and images for: Thoracic pedicle classification determined by inner cortical width of pedicles on computed tomography images: its clinical significance for posterior vertebral column resection to treat rigid and severe spinal deformities—a retrospective review of cases
Source: BMC Musculoskelet Disord. 2014 Aug 13;15:278. doi: 10.1186/1471-2474-15-278 (PMC4141956; doi:10.1186/1471-2474-15-278)

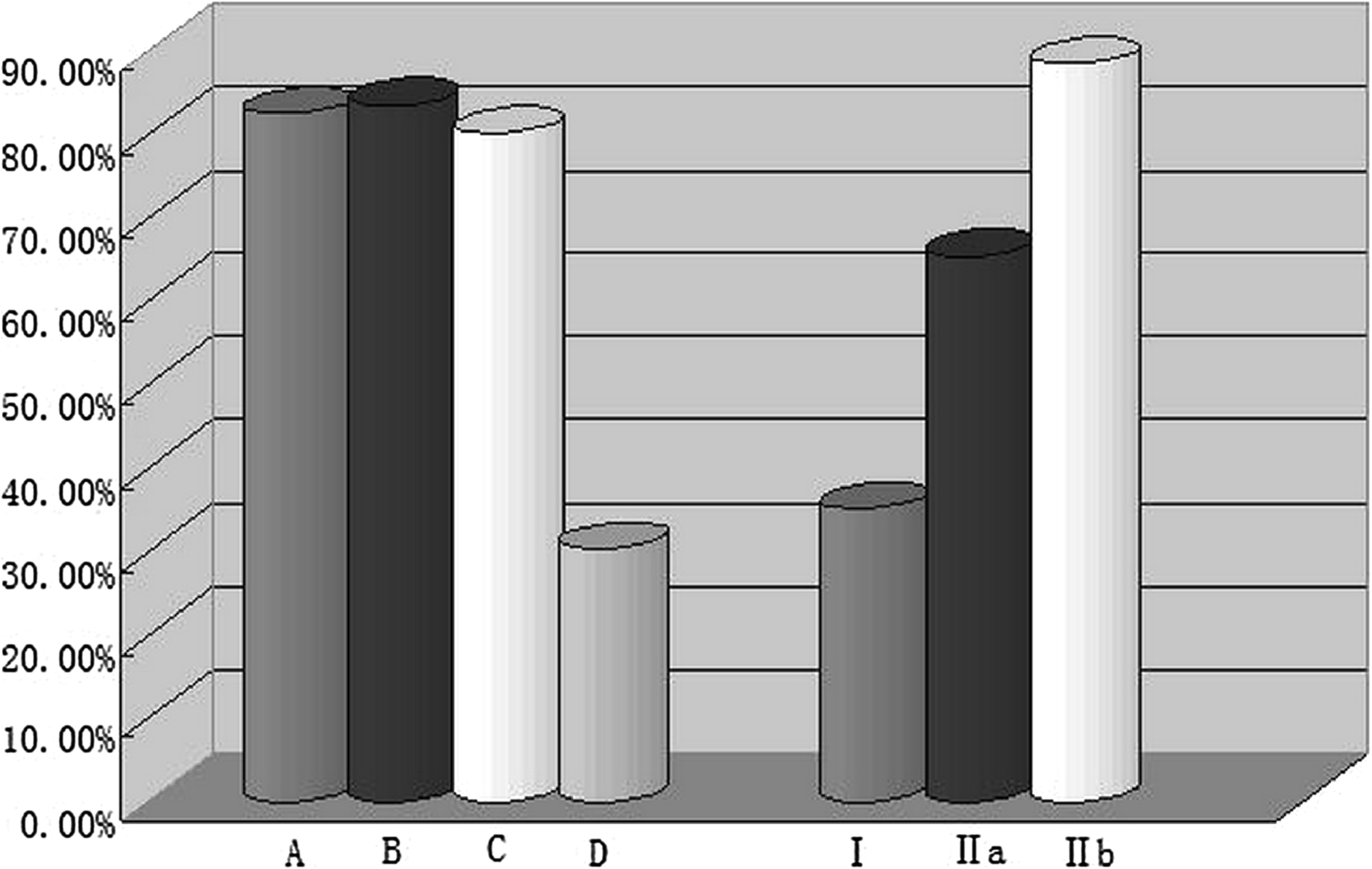

Supplement: Supplementary file 1 — Authors’ original file for figure 1 [file 12891_2014_2225_MOESM1_ESM.tiff]

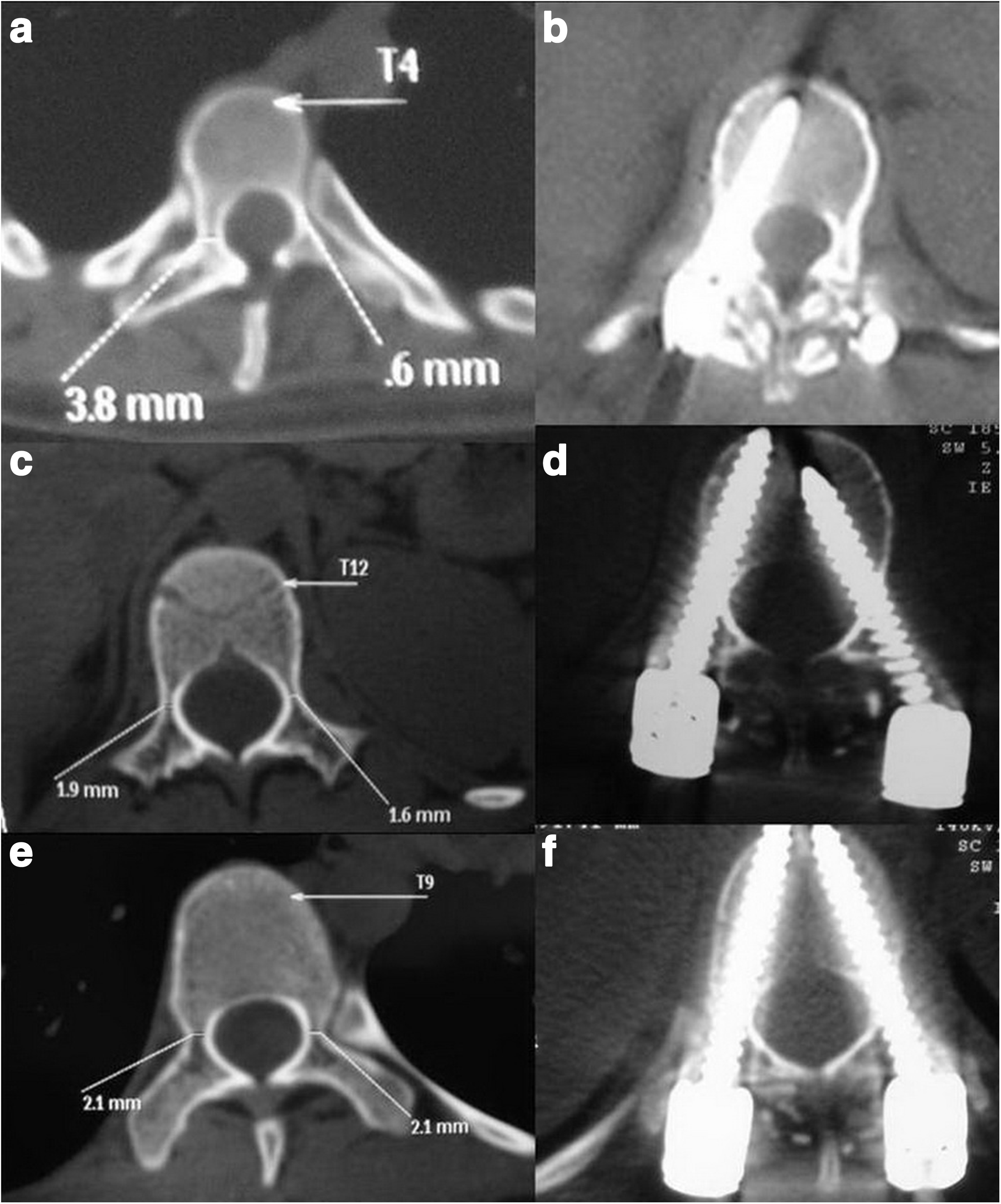

Supplement: Supplementary file 2 — Authors’ original file for figure 2 [file 12891_2014_2225_MOESM2_ESM.tiff]

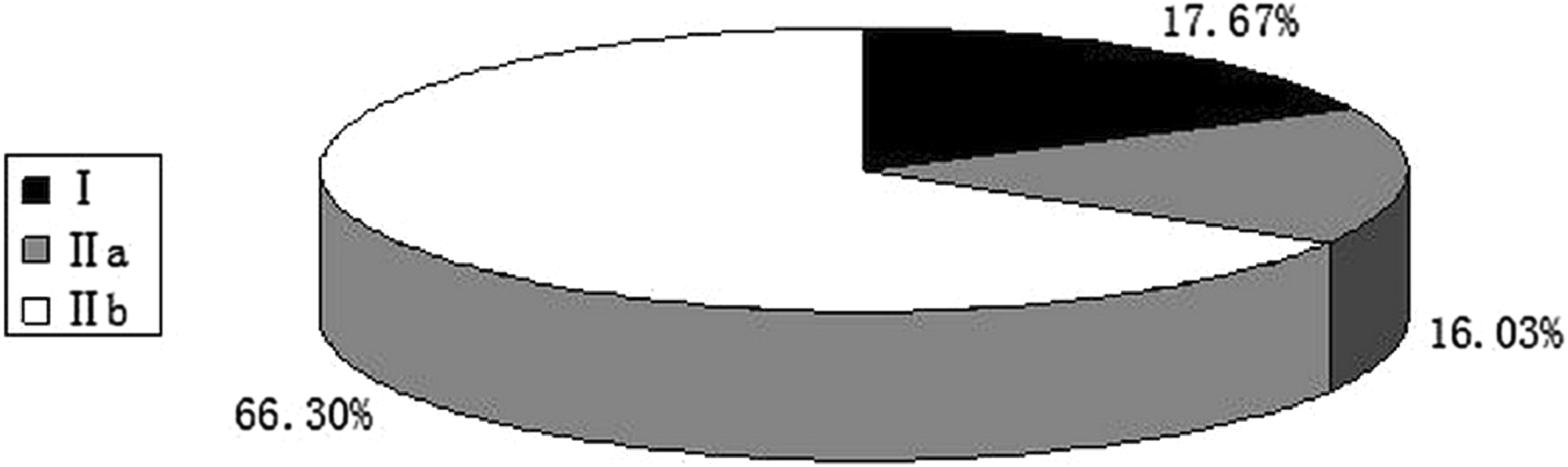

Supplement: Supplementary file 3 — Authors’ original file for figure 3 [file 12891_2014_2225_MOESM3_ESM.tiff]
